# The role of insulin resistance in the relationship between uric acid and the severity of coronary artery disease: evidence from real-world data

**DOI:** 10.3389/fnut.2025.1660317

**Published:** 2025-10-06

**Authors:** Zhongyin Chen, Lijing Shang, Ming Wang, Linfeng He

**Affiliations:** ^1^Department of Clinical Laboratory, Institute of Translational Medicine, Renmin Hospital of Wuhan University, Wuhan, Hubei, China; ^2^Department of General Medicine, Huazhong University of Science and Technology Hospital, Wuhan, Hubei, China; ^3^Department of Endocrinology, Union Hospital, Tongji Medical College, Huazhong University of Science and Technology, Wuhan, Hubei, China

**Keywords:** uric acid, coronary artery disease, insulin resistance, triglyceride-glucose index, mediation

## Abstract

**Background:**

The link between uric acid (UA) and cardiovascular diseases is debated, with insulin resistance possibly affecting this relationship. The triglyceride-glucose (TyG) index is a recognized marker for insulin resistance. However, the combined effect of different levels of UA and TyG on the severity of coronary artery disease (CAD) remains unclear.

**Methods:**

A cohort of 1,835 patients with newly diagnosed CAD was divided into single-vessel (743 patients) and multi-vessel (1,092 patients) CAD groups. The study utilized logistic regression, restricted cubic spline (RCS), and extreme gradient boosting (XGBoost) models to explore the associations between UA, TyG, and multi-vessel CAD. Interaction analysis assessed potential additive and multiplicative interactions. A mediation analysis was performed to assess the indirect effects of TyG and UA on the severity of CAD.

**Results:**

The TyG > 9.33 and non-HUA group is linked to a higher risk of multi-vessel CAD (OR 1.41, 95% CI 1.08–1.85), while the TyG ≤ 9.33 and HUA group shows no significant association (OR 1.08, 95% CI 0.76–1.53). The feature importance analysis, using the XGBoost model, demonstrated that TyG has a higher predictive value for multi-vessel CAD. No nonlinear correlations were observed for RCS. No notable additive or multiplicative interactions were detected between TyG and UA. Mediation analysis revealed that TyG significantly mediated the relationship between UA and multi-vessel CAD, with a proportion mediated of 18.89% (*p* = 0.026). In contrast, UA did not significantly mediate the TyG–CAD relationship (*p* = 0.082).

**Conclusion:**

The TyG index correlated more strongly with multi-vessel CAD compared to UA. Hyperuricemia correlated with multi-vessel CAD exclusively at elevated TyG levels, with TyG mediating the link between uric acid and CAD severity.

## 1 Introduction

Coronary artery disease (CAD) is a major global contributor to morbidity and mortality, significantly straining healthcare systems (1). Despite significant advances in diagnostic and therapeutic strategies, the complex pathophysiology of CAD continues to present challenges, particularly in assessing the severity of coronary artery lesions, which is a crucial determinant of patient outcomes (2, 3). Coronary angiography, though the definitive method for assessing coronary lesions, is not widely applicable due to its high cost and invasive nature, rendering it impractical for broad use, particularly among asymptomatic individuals. Understanding the multifactorial nature of CAD and its metabolic risk factors is crucial for identifying new prognostic markers and therapeutic targets in atherosclerotic lesion development and progression.

Growing evidence indicates a strong link between metabolic disorders like insulin resistance (IR) and hyperuricemia (HUA) and a heightened risk of CAD. Insulin resistance contributes to endothelial dysfunction, oxidative stress, and inflammation, which expedite atherosclerosis (4, 5). HUA contributes to cardiovascular disease pathogenesis by affecting endothelial function, oxidative stress, and inflammation (6). A recent study indicates that the combination of insulin resistance and hyperuricemia elevates the risk of major adverse cardiovascular events (MACE) in patients with glucose metabolism disorders (7). However, the precise mechanisms underlying the interplay between IR and HUA in the context of CAD remain to be fully elucidated.

The triglyceride-glucose (TyG) index has recently been recognized as a new and dependable surrogate marker for insulin resistance, showing a strong correlation with the euglycemic-hyperinsulinemic clamp, which is the gold standard for assessing insulin resistance. Research has shown a link between the TyG index and both the presence and severity of CAD (8, 9). The TyG index is a predictor of adverse cardiovascular events across different patient groups (10). The involvement of uric acid (UA) in cardiovascular diseases continues to be a topic of debate (11). Several studies have established a significant association between elevated serum UA levels and increased cardiovascular risk, including in patients with hypertension, where hyperuricemia is considered an independent risk factor for adverse outcomes (12, 13). For example, the uric acid right for heart health (Urrah) study by Muiesan et al. (14) and Mengozzi et al. (15) demonstrated that elevated UA levels were associated with increased cardiovascular risk in hypertensive patients, emphasizing the need for UA monitoring in this high-risk group. However, other studies have reported conflicting results, suggesting that the relationship between UA and cardiovascular risk may depend on other factors such as comorbidities, treatment regimens, and the presence of metabolic syndrome (16). Notably, a study combining a national cohort and meta-analysis confirmed an increased risk of cardiovascular mortality with high UA levels in diabetic patients, indicating that UA is inextricably linked to insulin resistance concerning cardiovascular disease (17).

Hence, the present study aims to investigate the combined effects of the TyG index and UA on the severity of coronary artery lesions in patients with newly diagnosed CAD. This study aims to offer new insights into the pathophysiological mechanisms connecting insulin resistance and hyperuricemia to the development and progression of atherosclerotic lesions by concentrating on this specific patient population.

## 2 Materials and methods

### 2.1 Study population

The study initially involved 2,533 patients diagnosed with CAD at a tertiary medical center from July 2009 to August 2011 (16). Eligible patients were those who had undergone coronary angiography without a prior history of coronary artery disease (CAD), percutaneous coronary intervention (PCI), or coronary artery bypass grafting (CABG). Participants were excluded if they had incomplete data for age, fasting plasma glucose (FPG), triglyceride (TG), and UA values (Supplementary Figure S1).

### 2.2 Definitions of CAD and multi-vessel CAD

Coronary angiographic analyses, both qualitative and quantitative, were conducted using established methods (16). CAD was characterized by the presence of at least one coronary artery stenosis of 50% or greater, as determined by coronary angiography (17). Multi-vessel CAD was characterized by significant stenosis (≥50%) in two or more major coronary arteries, such as the left anterior descending (LAD), left circumflex (LCX), or right coronary artery (RCA). Lesions in the left main coronary artery (LMCA) were classified as multi-vessel CAD. Patients were categorized into single-vessel CAD and multi-vessel CAD groups according to the number of affected vessels.

### 2.3 TyG index calculation

The TyG index, a dependable marker for insulin resistance, was derived from TG and FPG measurements. The TyG calculation is determined using the following formula.

TyG index = ln [FPG(mg/dl) × TG(mg/dl)/2]

### 2.4 TyG and UA classification

The receiver operating characteristic (ROC) curve analysis determined the optimal TyG index cutoff value to be 9.33 (Supplementary Figure S2). Patients were divided into two groups based on TyG values: those with TyG > 9.33 and those with TyG ≤ 9.33. Patients were categorized into HUA and non-HUA groups based on diagnostic criteria, where HUA is defined by serum uric acid levels exceeding 7 mg/dL in men and 6 mg/dL in women (18). Consequently, the study population was further divided into four groups based on combined TyG and UA levels: TyG ≤ 9.33 and non-HUA, TyG > 9.33 and non-HUA, TyG ≤ 9.33 and HUA, and TyG > 9.33 and HUA.

### 2.5 Data collection

Demographic and clinical data, including age, sex, BMI, smoking status, and history of comorbidities like hypertension and diabetes, were obtained from the hospital information database. Laboratory data, including FPG, TG, UA, and lipid profiles, were measured using standard biochemical assays. Renal function was assessed using the estimated glomerular filtration rate (eGFR) calculated with the CKD-EPI formula. Coronary angiography findings were reviewed by experienced cardiologists who assessed the severity and extent of CAD (1).

### 2.6 Statistical analysis

Statistical analyses were performed using R software (v4.3.0) and Empower software (v4.1). The normality of continuous variables was assessed using the Kolmogorov–Smirnov test. Data that passed the normality test (*p* > 0.05) were expressed as the mean ± standard deviation (SD) and compared using *t*-tests. Data that did not meet the normality assumption were presented as the median ± interquartile range (IQR), and comparisons were made using the Mann–Whitney *U* test. Categorical variables were summarized as frequencies and percentages and analyzed using chi-square tests. A logistic regression analysis evaluated the association between the TyG index, UA, and the risk of multi-vessel CAD. Collinearity diagnosis was performed, indicating that the level of collinearity among the variables was within acceptable limits, as evidenced by all variance inflation factors (VIF) being less than 5 (Supplementary Table S1). A restricted cubic spline (RCS) analysis was conducted to evaluate the linearity between the TyG index, UA, and multi-vessel CAD. The significance of TyG and UA in multi-vessel CAD was evaluated using extreme gradient boosting (XGBoost) and Shapley additive explanations (SHAP) values. Interaction and mediation effects were assessed using additive and multiplicative interaction models and mediation analysis. Statistical significance was defined as a *p*-value less than 0.05.

## 3 Results

### 3.1 Baseline demographic and clinical characteristics

In this study involving 1,835 newly diagnosed CAD patients, notable demographic and clinical differences were found between individuals with single-vessel and multi-vessel CAD. The multi-vessel CAD group had an older average age of 60.97 years, compared to 57.64 years in the single-vessel group (*p* < 0.001). The groups showed no significant difference in sex distribution. Blood pressure measurements and heart rate showed no significant differences. FPG levels were notably elevated in the multi-vessel CAD group (*p* < 0.001), suggesting a link between impaired glucose metabolism and increased severity of coronary artery involvement. Serum TG levels were marginally higher in the multi-vessel group (*p* = 0.049).

The multi-vessel group exhibited higher low-density lipoprotein cholesterol (LDL-C) levels (*p* = 0.016) and a significantly elevated TyG index (*p* < 0.001). The multi-vessel CAD group exhibited significantly elevated UA levels (*p* = 0.037), indicating a potential association between hyperuricemia and extensive coronary disease. Comorbidities such as heart failure (*p* = 0.003), hypertension (*p* = 0.048), and diabetes (*p* < 0.001) were more common in individuals with multi-vessel CAD. Patients in the multi-vessel group exhibited a higher incidence of ST-segment elevation myocardial infarction (*p* = 0.031), suggesting more severe clinical presentations. The results indicate that metabolic dysfunction, especially concerning glucose and lipid metabolism, combined with elevated UA levels, may contribute to the progression and severity of CAD (Table 1).

**Table 1:** Characteristics of study subjects grouped according to severity of coronary lesions.

Characteristics	Single-vessel CAD	Multi-vessel CAD	SMD	*p*-value
*N*	743	1,092		
Age, years	57.64 (11.20)	60.97 (10.81)	0.30 (0.21, 0.40)	<0.001
Sex			0.01 (−0.08, 0.11)	0.795
Men	252 (33.92%)	364 (33.33%)		
Women	491 (66.08%)	728 (66.67%)		
SBP, mmHg	103.32 (27.73)	101.81 (29.00)	0.05 (−0.04, 0.15)	0.266
DBP, mmHg	77.38 (11.61)	77.17 (12.24)	0.02 (−0.08, 0.11)	0.715
BMI classification			0.10 (0.01, 0.20)	0.208
<24 kg/m^2^	272 (36.61%)	398 (36.45%)		
24–28 kg/m^2^	149 (20.05%)	259 (23.72%)		
≥28 kg/m^2^	71 (9.56%)	106 (9.71%)		
Missing data	251 (33.78%)	329 (30.13%)		
Heart rate, bpm	72.32 (11.44)	72.11 (11.76)	0.02 (−0.08, 0.11)	0.714
FPG, mmol/L	5.10 (4.62–6.01)	5.30 (4.73–6.56)	0.14 (0.05, 0.24)	<0.001
TG, mmol/L	1.53 (1.13–2.20)	1.65 (1.17–2.35)	0.10 (0.01, 0.20)	0.049
HDL-C, mmol/L	1.06 (0.31)	1.06 (0.31)	0.01 (−0.09, 0.10)	0.863
LDL-C, mmol/L	2.65 (0.91)	2.75 (0.95)	0.12 (0.02, 0.21)	0.016
eGFR, ml/(min·1.73 m^2^)	99.71 (19.68)	95.05 (18.85)	0.24 (0.15, 0.34)	<0.001
UA, mg/dl	4.97 (1.50)	5.13 (1.62)	0.10 (0.01, 0.19)	0.037
TyG	8.85 (0.63)	8.96 (0.67)	0.18 (0.08, 0.27)	<0.001
CAD classification			0.13 (0.03, 0.22)	0.031
ST-segment elevation myocardial infarction	184 (24.76%)	332 (30.40%)		
Non-ST-segment elevation myocardial infarction	487 (65.55%)	664 (60.81%)		
Stable CAD	72 (9.69%)	96 (8.79%)		
Location of diseased coronary artery				
LMCA	0 (0.00%)	59 (5.40%)	0.34 (0.24, 0.43)	<0.001
LAD	532 (71.60%)	988 (90.48%)	0.50 (0.40, 0.59)	<0.001
LCX	93 (12.52%)	788 (72.16%)	1.53 (1.42, 1.63)	<0.001
RCA	118 (15.88%)	766 (70.15%)	1.31 (1.21, 1.41)	<0.001
History of disease				
Heart failure	66 (8.91%)	147 (13.47%)	0.15 (0.05, 0.24)	0.003
Atrial fibrillation	12 (1.62%)	23 (2.11%)	0.04 (−0.06, 0.13)	0.45
Cardiac shock	1 (0.13%)	2 (0.18%)	0.01 (−0.08, 0.11)	0.999
COPD	6 (0.81%)	12 (1.10%)	0.03 (−0.06, 0.12)	0.534
Stroke	35 (4.71%)	59 (5.40%)	0.03 (−0.06, 0.12)	0.509
Hypertension	421 (56.74%)	670 (61.36%)	0.09 (0.00, 0.19)	0.048
Diabetes	206 (27.76%)	423 (38.74%)	0.23 (0.14, 0.33)	<0.001
Smoking	229 (30.82%)	364 (33.33%)	0.05 (−0.04, 0.15)	0.259

Data are expressed as mean (SD), median (IQR) or *n* (%).

CAD, coronary artery disease; SMD, standardized mean difference; SD, standard deviation; IQR, interquartile range; SBP, systolic blood pressure; DBP, diastolic blood pressure; BMI, body mass index; FPG, fasting plasma glucose; TG, triglyceride; HDL-C, high-density lipoprotein cholesterol; LDL-C, low-density lipoprotein cholesterol; eGFR, estimated glomerular filtration rate; UA, uric acid; TyG, triglyceride glucose index; LAD, left anterior descending; LCX, left circumflex; RCA, right coronary artery. LMCA, lesions in the left main coronary artery; COPD, chronic obstructive pulmonary disease.

### 3.2 Relationship between TyG index, UA, and multi-vessel CAD

Logistic regression analysis revealed that both the TyG index and UA levels were independently associated with an increased risk of multi-vessel CAD. Patients with a TyG index > 9.33 had a higher risk, which was further elevated in those with HUA. In Model 2, patients with TyG > 9.33 and HUA had nearly double the risk of multi-vessel CAD compared to those with lower TyG and no HUA (OR 1.86, 95% CI 1.08–3.21; Table 2), while those with low TyG and HUA showed no significant difference (OR 1.08, 95% CI 0.76–1.53).

**Table 2:** Logistic regression analysis for multi-vessel CAD.

Variables	OR (95%CI)		
	Crude model	Model 1	Model 2
TyG (per unit)	1.31 (1.13 ~ 1.51)	1.41 (1.22 ~ 1.64)	1.27 (1.08 ~ 1.5)
TyG (per SD)	1.19 (1.08 ~ 1.31)	1.26 (1.14 ~ 1.39)	1.17 (1.05 ~ 1.31)
TyG ≤ 9.33	Reference	Reference	Reference
TyG > 9.33	1.57 (1.25 ~ 1.97)	1.69 (1.34 ~ 2.13)	1.47 (1.14 ~ 1.89)
UA (per unit)	1.07 (1 ~ 1.13)	1.06 (1 ~ 1.13)	1.08 (1.01 ~ 1.15)
UA (per SD)	1.11 (1.01 ~ 1.22)	1.1 (1 ~ 1.22)	1.12 (1.01 ~ 1.24)
Non-HUA	Reference	Reference	Reference
HUA	1.27 (0.95 ~ 1.7)	1.21 (0.9 ~ 1.63)	1.19 (0.88 ~ 1.6)
TyG ≤ 9.33 and Non-HUA	Reference	Reference	Reference
TyG > 9.33 and Non-HUA	1.54 (1.2 ~ 1.97)	1.65 (1.28 ~ 2.12)	1.41 (1.08 ~ 1.85)
TyG ≤ 9.33 and HUA	1.2 (0.85 ~ 1.68)	1.12 (0.79 ~ 1.59)	1.08 (0.76 ~ 1.53)
TyG > 9.33 and HUA	2 (1.18 ~ 3.38)	2.05 (1.2 ~ 3.5)	1.86 (1.08 ~ 3.21)

Model 1: adjusted for age and sex.

Model 2: adjusted for Model 1, SBP, DBP, BMI, CAD classification, heart failure, atrial fibrillation, COPD, stroke, diabetes, and smoking.

OR odds ratio, CI confidence interval, HUA hyperuricemia, other abbreviations can be found in Table 1.

**Figure 1:**
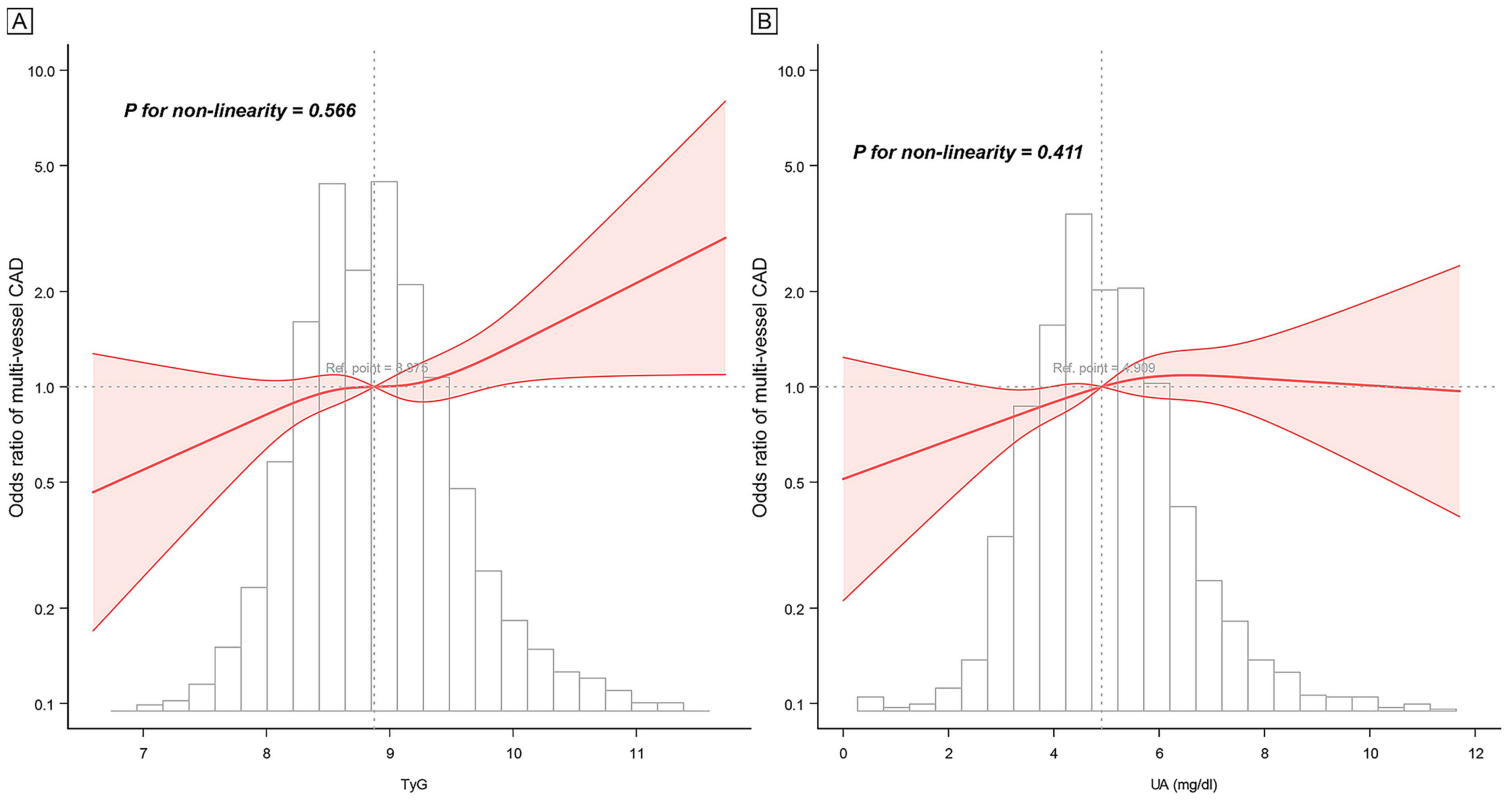
Restricted cubic spline curve of TyG, UA, and multi-vessel CAD. **(A)** TyG, **(B)** UA. Adjusted for all covariates in model 2.

The RCS analysis demonstrated that the relationships between TyG, UA, and multi-vessel CAD followed a linear pattern (Figure 1). This indicates a continuous increase in the risk of multi-vessel CAD with rising TyG and UA levels, without any thresholds or inflection points. Subgroup analyses indicated that the relationship between TyG, UA, and multi-vessel CAD remained consistent across diverse populations, including different age groups, sexes, BMI groups, and clinical conditions like hypertension, diabetes, and CAD types (all *P* for interaction > 0.05, Supplementary Figure S3 and Supplementary Table S3). The consistency of these findings across diverse patient groups underscores the robustness of TyG and UA as predictors of CAD severity.

### 3.3 Feature importance and impact on multi-vessel CAD

**Figure 2:**
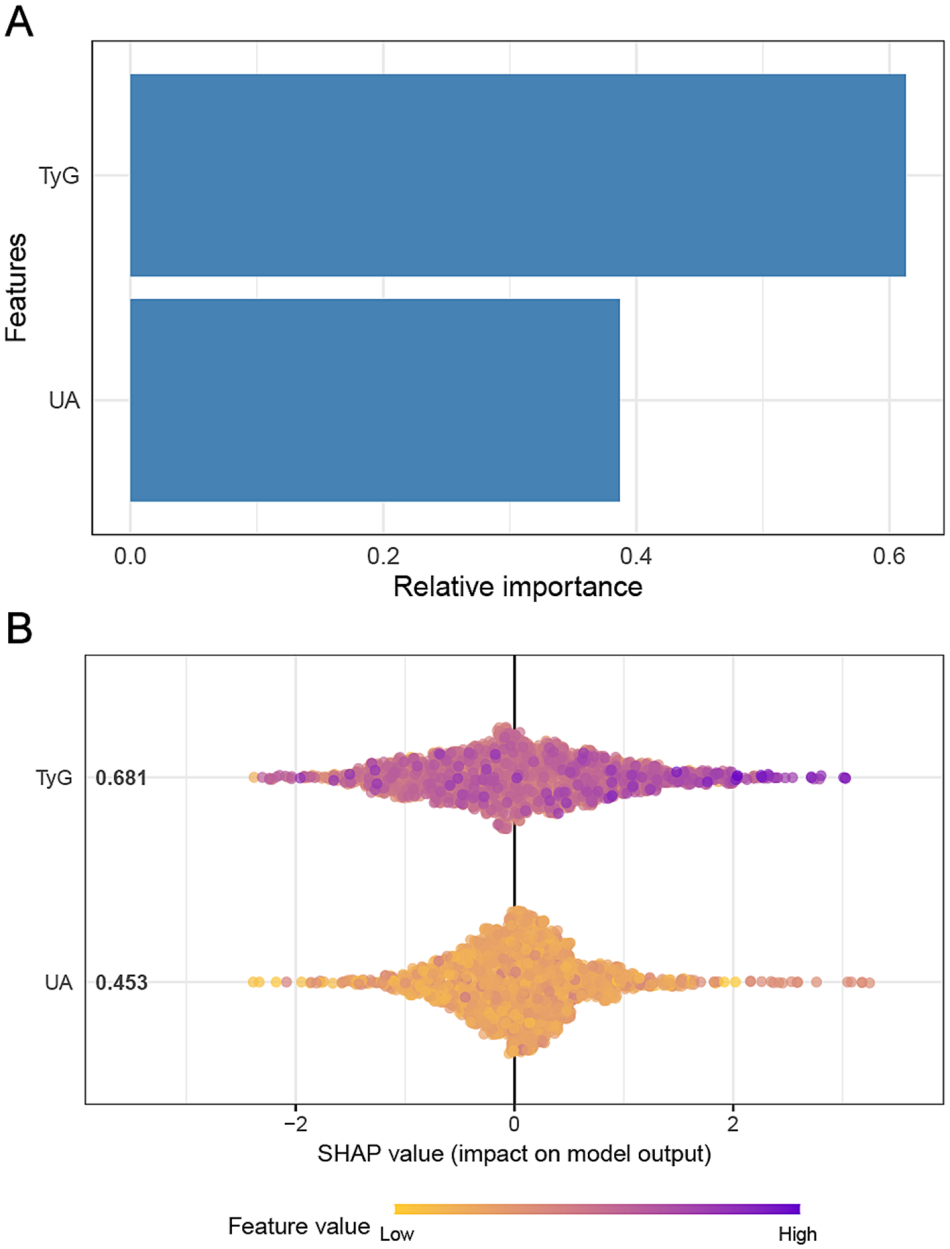
Impact of TyG and UA on multi-vessel CAD based on XGBoost model. Panel **(A)** illustrates the relative importance of the features in predicting multi-vessel CAD using the XGBoost model. Panel **(B)** presents the SHAP values.

The analysis of feature importance in the prediction of multi-vessel CAD, using the XGBoost model, revealed that the TyG holds a more substantial predictive power than UA. As shown in Figure 2A, TyG demonstrates a higher relative importance compared to UA, highlighting its stronger association with CAD severity. In Figure 2B, the SHAP values further support this finding, indicating that higher TyG values exert a more significant influence on model predictions. The SHAP plot visualizes the impact of TyG and UA on multi-vessel CAD severity, with the color gradient representing varying feature values, from low (yellow) to high (purple). This indicates that both TyG and UA contribute meaningfully to the model’s output, though TyG appears to play a more prominent role in determining multi-vessel CAD severity.

### 3.4 Interaction and mediation of TyG and UA

The study found no significant additive or multiplicative interaction effects between TyG, UA, and multi-vessel CAD. The synergy index (SI) suggested a potential additive interaction (SI = 1.75, 95% CI = 0.37–8.18, *p* = 0.02), whereas the relative excess risk due to interaction (RERI) and attributable proportion (AP) were not statistically significant (*p* = 0.25 and *p* = 0.22, respectively). The multiplicative interaction was also non-significant (*p* = 0.56), suggesting limited interaction between TyG and UA regarding multi-vessel CAD risk (Table 3).

**Table 3:** Interaction analysis of TyG, UA, and multi-vessel CAD.

Parameters	Value (95% CI)	*p*-value
Additive interaction		
RERI	0.37 (−0.73 ~ 1.47)	0.25
AP	0.2 (−0.3 ~ 0.7)	0.22
SI	1.75 (0.37 ~ 8.18)	0.02
Multiplicative interaction	1.22 (0.62 ~ 2.4)	0.56

CI, confidence interval; RERI, relative excess risk interacting; AP, attributable proportion due to interaction; SI, synergy index, other abbreviations can be found in Table 1.

**Figure 3:**
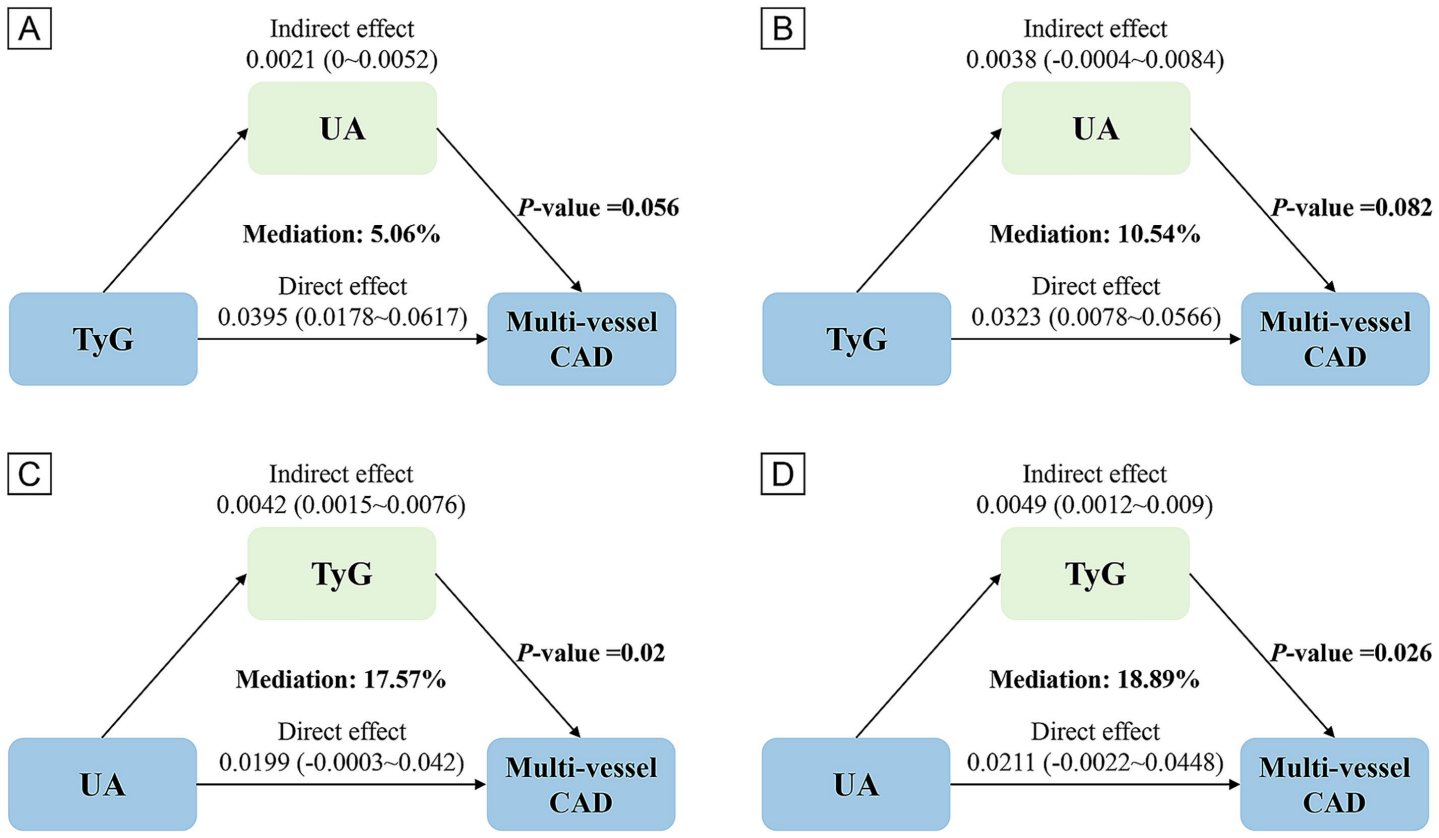
Analysis of the mediating role of TyG and UA. Panels **(A,C)** based on Crude model, **(B,D)** based on Model 2.

The UA did not significantly mediate the TyG and multi-vessel CAD relationship (10.54% mediated, *p* = 0.082). However, TyG significantly mediated the relationship between UA and multi-vessel CAD, with a proportion mediated of 18.89% (*p* = 0.026), indicating that TyG explains the effect of UA on multi-vessel CAD risk (Figure 3 and Supplementary Table S2).

## 4 Discussion

This study aimed to explore the associations between the TyG index, UA, and CAD severity in a Chinese cohort. Our study identifies strong correlations between a high TyG index, elevated UA levels, and an increased risk of multi-vessel CAD. Our findings indicate that the TyG index mediates the impact of UA on CAD severity, offering new insights into the intricate relationship between these metabolic markers and cardiovascular risk.

The study indicates a significant correlation between elevated TyG levels and a heightened risk of multi-vessel CAD, with each unit increase in the TyG index corresponding to an odds ratio of 1.27 (95% CI 1.08–1.50). This finding is consistent with recent research emphasizing the TyG index as an indicator of insulin resistance and its potential in forecasting cardiovascular events. For instance, a large-scale study by Xiong et al. (19). A study involving 1,007 individuals demonstrated that a higher TyG index correlates with an increased SYNTAX score in acute coronary syndrome patients. Similarly, Liang et al. (10) a meta-analysis of studies found that an elevated TyG index independently correlates with an increased risk of CAD and more severe coronary lesions. Various mechanisms may account for the observed link between the TyG index and multi-vessel CAD. The TyG index serves as a surrogate marker for insulin resistance, indicating disruptions in glucose and lipid metabolism. Insulin resistance has been shown to promote endothelial dysfunction, vascular inflammation, and the progression of atherosclerosis (5). Li et al. (20) conducted a recent study. An elevated TyG index was linked to increased arterial stiffness, with lipids and inflammation partially mediating this relationship.

Our study identified a significant link between high UA levels and a heightened risk of multi-vessel CAD. This relationship has been corroborated by recent research, including a Mendelian randomization study by Zhang et al. (21). The study, involving 343,836 participants, revealed a dose–response relationship between serum UA levels and CAD risk (21). The mechanisms linking UA to CAD progression are multifaceted and include promoting oxidative stress, endothelial dysfunction, and vascular inflammation (22).

Our findings indicate that the TyG index and UA may play complementary roles in evaluating cardiovascular risk. While both markers were independently associated with multi-vessel CAD, their combined presence further amplified the risk. This synergistic effect underscores the importance of considering multiple metabolic parameters when evaluating cardiovascular risk. A recent study by Wu et al. (7) found that the combination of elevated TyG index and UA levels was associated with a higher risk of adverse cardiovascular outcomes in patients receiving CABG, supporting our observations. The potential mechanisms linking the TyG index and UA to CAD severity extend beyond their individual effects. Both markers are closely related to metabolic dysfunction and insulin resistance. Recent studies indicate that insulin resistance may intensify the pro-inflammatory and pro-oxidative impacts of UA (23). Hyperuricemia can lead to insulin resistance by disrupting insulin signaling and glucose uptake in skeletal muscle (24).

Our analysis found no significant additive or multiplicative interactions between the TyG index and UA concerning multi-vessel CAD risk. While this suggests that the effects of these markers on CAD risk may be largely independent, the observed significant synergy index (SI) value of 0.02 warrants further consideration. This seemingly contradictory finding—where SI is significant but relative excess risk due to interaction (RERI) and attributable proportion (AP) are not—could be attributed to the complex interplay between insulin resistance and hyperuricemia in the pathogenesis of CAD. It is possible that the lack of significant interaction reflects the limitations in capturing the full scope of biological and clinical interactions, particularly given the absence of data on drug use that might affect UA levels or insulin sensitivity. The lack of a significant interaction in our study contrasts with previous research, such as that by Wu et al. (7), who identified a significant interaction between TyG and UA in predicting adverse cardiovascular outcomes in patients undergoing coronary artery bypass grafting (CABG). Differences in study populations, such as the presence of comorbidities or treatment regimens, and variations in outcomes of interest, may explain these conflicting results. Furthermore, the statistical power in our study might have been insufficient to detect subtle interactions, particularly given the large sample size and the complexity of the potential interactions. Therefore, while our findings suggest no significant additive or multiplicative interactions, it is essential to interpret this result within the context of the study’s limitations, including the lack of direct measurement of drug effects.

Despite the lack of significant interaction, our mediation analysis revealed a crucial finding: the TyG index significantly mediates the relationship between UA and multi-vessel CAD, with a proportion mediated of 18.89% (*p* = 0.026). This novel observation suggests that a substantial portion of the effect of UA on CAD severity may be explained by its influence on glucose and lipid metabolism, as reflected by the TyG index. Research in Xinjiang, China, revealed that patients undergoing percutaneous coronary intervention (PCI) exhibited significantly elevated UA levels compared to individuals with normal coronary angiograms (25). This study concluded that while UA was not an independent risk factor for CAD severity, it was linked to other cardiometabolic risk factors like cholesterol and triglycerides, supporting our findings. The role of TyG as a mediator in the UA-CAD pathway is crucial for understanding how metabolic dysfunction connects to cardiovascular disease. It suggests that, at least partially, UA may exert its pro-atherogenic effects by promoting insulin resistance and dyslipidemia. Recent experimental studies support this finding. Zhu et al. (26) demonstrated that UA can induce insulin resistance in adipocytes by activating the NLRP3 inflammasome, leading to impaired insulin signaling and reduced glucose uptake. Furthermore, Lanaspa et al. (27) UA has been demonstrated to enhance hepatic lipogenesis and elevate triglyceride production by activating the carbohydrate-responsive element-binding protein. Identifying TyG as a mediator in the UA-CAD relationship provides new insights into potential therapeutic targets. Interventions to improve insulin sensitivity and lipid metabolism may be particularly effective in mitigating the cardiovascular risk associated with hyperuricemia. Recent clinical trials support this concept. Zhao et al. (28) conducted a meta-analysis of 62 randomized controlled trials with 34,941 participants, revealing that sodium-glucose cotransporter 2 (SGLT2) inhibitors enhance glycemic control and lower serum uric acid levels in type 2 diabetes patients. SGLT2 inhibitors may partly improve endothelial function by lowering UA levels to reduce insulin resistance (29).

Our findings have some significant clinical implications. The TyG index’s significant link to multi-vessel CAD and its intermediary function in the UA-CAD pathway underscore its potential as an efficient and economical tool for CAD risk assessment. Recent research highlights the TyG index’s enhanced contribution to cardiovascular risk prediction models. For instance, Pang et al. (30) incorporating the TyG index into the GRACE Score significantly improved its predictive accuracy for future cardiovascular events. The combined TyG index and UA levels assessment offers a more comprehensive approach to identifying high-risk individuals. Our findings suggest that individuals with elevated TyG and UA levels face a heightened risk of multi-vessel CAD, supporting the trend toward multi-marker strategies in cardiovascular risk evaluation (31). These insights may influence patient management and prevention strategies by emphasizing the importance of addressing insulin resistance and metabolic dysfunction in CAD prevention. Lifestyle interventions targeting insulin sensitivity and pharmacological interventions that improve insulin sensitivity or lower UA levels may be particularly beneficial in reducing cardiovascular risk.

Despite its strengths, our study has several limitations. The cross-sectional design precludes the establishment of causal relationships between the TyG index, UA, and multi-vessel CAD. Our mediation analysis indicates a possible causal pathway, but longitudinal studies are necessary to validate these relationships and determine the temporal sequence of events. Residual confounding may persist even after adjusting for various factors. Unconsidered variables like proteinuria, albumin-creatinine ratio, dietary habits, physical activity, and genetic predisposition may affect both metabolic markers and CAD risk. The lack of information on medication use, particularly drugs affecting UA levels or insulin sensitivity, is another limitation. Further research is needed to determine if our findings apply to other populations. Recent studies across various ethnic groups suggest that the biological mechanisms linking the TyG index, UA, and CAD are likely applicable to other populations (32–34).

## 5 Conclusion

In conclusion, insulin resistance significantly influences the association of UA with the severity of CAD in a Chinese population. The discovery that the TyG index mediates the impact of UA on CAD severity offers fresh insights into the intricate relationship between these metabolic markers and cardiovascular risk.

## Data Availability

Publicly available datasets were analyzed in this study. This data can be found: Dryad repository (https://datadryad.org).

## Glossary

AP: attributable proportion
BMI: body mass index
CAD: coronary artery disease
CABG: coronary artery bypass grafting
CI: confidence interval
COPD: chronic obstructive pulmonary disease
DBP: diastolic blood pressure
eGFR: estimated glomerular filtration rate
FPG: fasting plasma glucose
HDL-C: high-density Lipoprotein Cholesterol
HUA: hyperuricemia
IQR: interquartile range
LDL-C: low-density Lipoprotein Cholesterol
MACE: major adverse cardiovascular events
NLRP3: NOD-like receptor protein 3
OR: odds ratio
PCI: percutaneous coronary intervention
RCS: restricted cubic spline
RERI: relative excess risk due to interaction
SBP: systolic blood pressure
SD: standard deviation
SGLT2: sodium-glucose cotransporter 2
SHAP: Shapley additive explanations
SI: synergy index
SMD: standardized mean difference
SYNTAX: synergy between PCI with Taxus and cardiac surgery
TG: triglyceride
TyG: triglyceride-glucose
UA: uric acid
Urrah: uric acid right for heart health
XGBoost: extreme gradient boosting
