# Supplementary material for: The role of insulin resistance in the relationship between uric acid and the severity of coronary artery disease: evidence from real-world data
Source: Front Nutr. 2025 Oct 6;12:1660317. doi: 10.3389/fnut.2025.1660317 (PMC12535908; doi:10.3389/fnut.2025.1660317)
Supplement: Supplementary file 1 [file Table_1.docx]

Supplementary Material

Table S1 Collinearity

test of covariables

|  | VIF |
| --- | --- |
| Age, years | 1.1 |
| Sex | 1.4 |
| SBP | 1.8 |
| DBP | 1.3 |
| BMI | 1.5 |
| CAD classification | 1.1 |
| UA | 1.1 |
| TyG | 1.5 |
| Heart failure | 1 |
| Atrial fibrillation | 1 |
| COPD | 1 |
| Stroke | 1 |
| Diabetes | 1.4 |
| Smoking | 1.3 |

VIF variance inflation factor

|  | Value (95% CI) | | | |
| --- | --- | --- | --- | --- |
|  | Crude model | | Adjusted model | |
| Exposure: TyG, Mediator: UA |  |  |  |  |
| Average Causal Mediation Effects | 0.0021 (0~0.0052) | 0.056 | 0.0038 (-0.0004~0.0084) | 0.078 |
| Average Direct Effects | 0.0395 (0.0178~0.0617) | < 0.001 | 0.0323 (0.0078~0.0566) | 0.006 |
| Total Effect | 0.0416 (0.0209~0.0639) | < 0.001 | 0.0361 (0.0116~0.0611) | 0.004 |
| Proportion of Mediated | 0.0506 (-0.0006~0.1513) | 0.056 | 0.1054 (-0.0128~0.396) | 0.082 |
| Exposure: UA, Mediator: TyG |  |  |  |  |
| Average Causal Mediation Effects | 0.0042 (0.0015~0.0076) | < 0.001 | 0.0049 (0.0012~0.009) | 0.006 |
| Average Direct Effects | 0.0199 (-0.0003~0.042) | 0.056 | 0.0211 (-0.0022~0.0448) | 0.078 |
| Total Effect | 0.0241 (0.004~0.0466) | 0.02 | 0.026 (0.0028~0.0499) | 0.02 |
| Proportion of Mediated | 0.1757 (0.0445~0.8459) | 0.02 | 0.1889 (0.0306~1.2393) | 0.026 |

Table S2 Analysis of the mediating role of TyG, uric acid, and multi-vessel CAD

Adjusted model adjusted for all covariates in model 2. CI confidence interval, other abbreviations can be found in Table 1.

**Table S3 BMI subgroup analysis of TyG, UA and multi-vessel CAD**

| **BMI groups** | **OR (95% CI)** | ***P* for interaciton** |
| --- | --- | --- |
|  |  | 0.538 |
| **BMI < 24 kg/m^2^** |  |  |
| TyG≤ 9.33 & Non-HUA | Reference |  |
| TyG> 9.33 & Non-HUA | 1.71 (1.09~2.66) |  |
| TyG≤ 9.33 & HUA | 1.43 (0.83~2.47) |  |
| TyG> 9.33 & HUA | 1.87 (0.73~4.78) |  |
| **BMI ≥ 24 kg/m^2^** |  |  |
| TyG≤ 9.33 & Non-HUA | Reference |  |
| TyG> 9.33 & Non-HUA | 1.32 (0.94~1.83) |  |
| TyG≤ 9.33 & HUA | 0.93 (0.59~1.50) |  |
| TyG> 9.33 & HUA | 2.04 (1.05~3.99) |  |

Abbreviations can be found in Table 1.





**Figure. S1** Flowchart of study subjects.


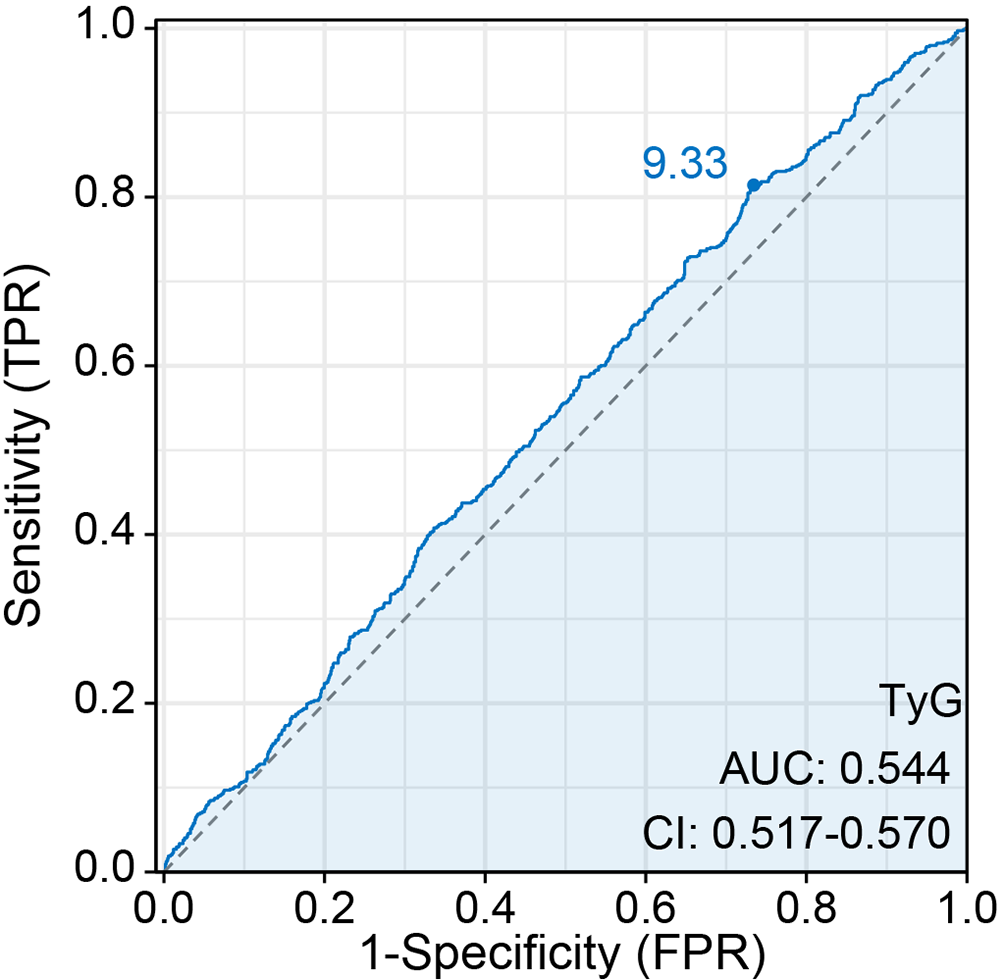


**Fig. S2** ROC analysis of TyG and multivessel CAD. Abbreviations can be found in Table 1.


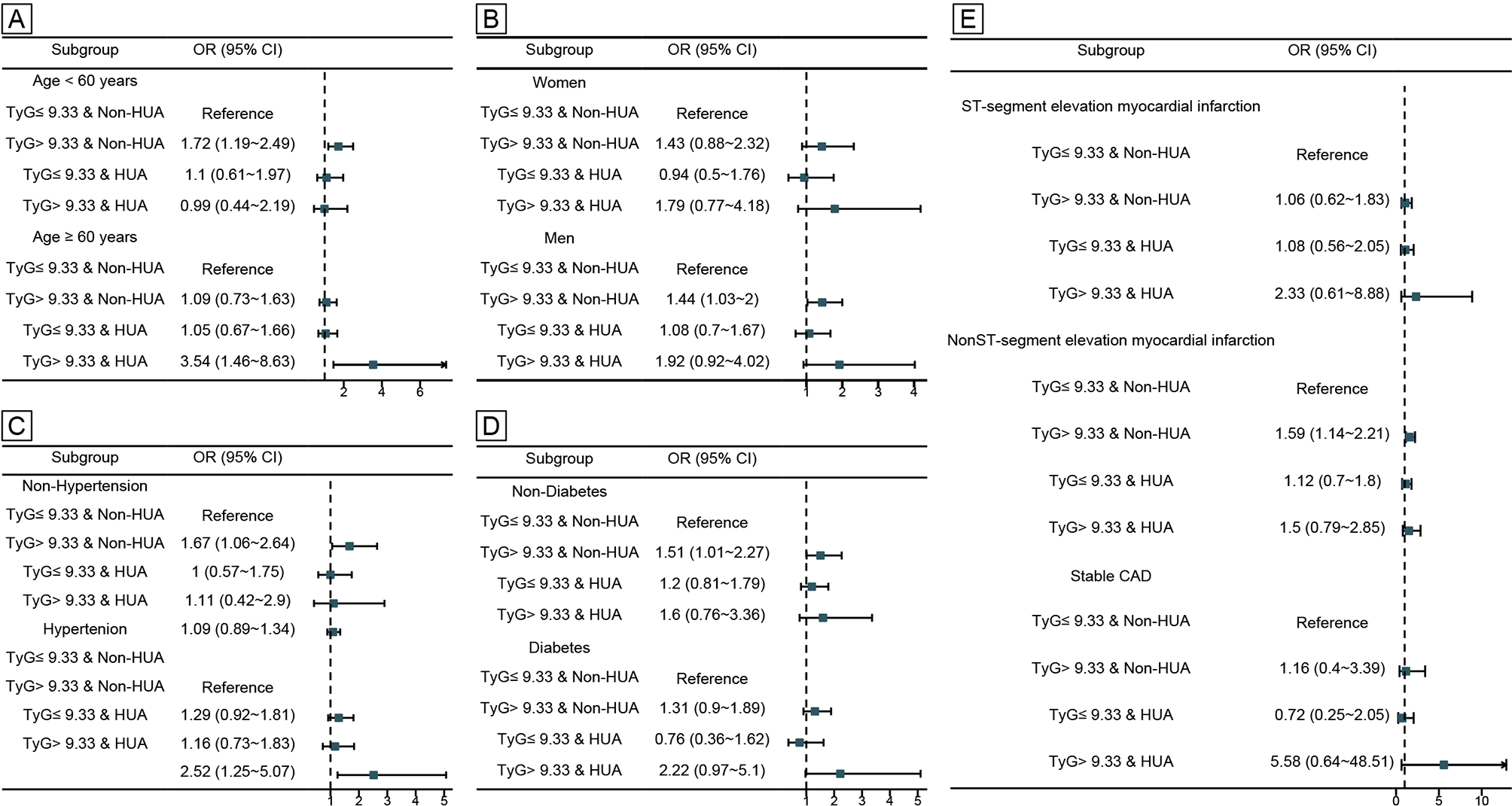


**Fig. S3** Subgroup analysis of TyG, UA and multi-vessel CAD. Abbreviations can be found in Table 1.
